# Supplementary material for: Potentially Pathogenic SORL1 Mutations Observed in Autosomal-Dominant Cases of Alzheimer’s Disease Do Not Modulate APP Physiopathological Processing
Source: Cells. 2023 Dec 8;12(24):2802. doi: 10.3390/cells12242802 (PMC10742224; doi:10.3390/cells12242802)
Supplement: Supplementary file 1 [file cells-12-02802-s001.zip › cells-2665629-SM/cells-2665629-supplementary.pdf]

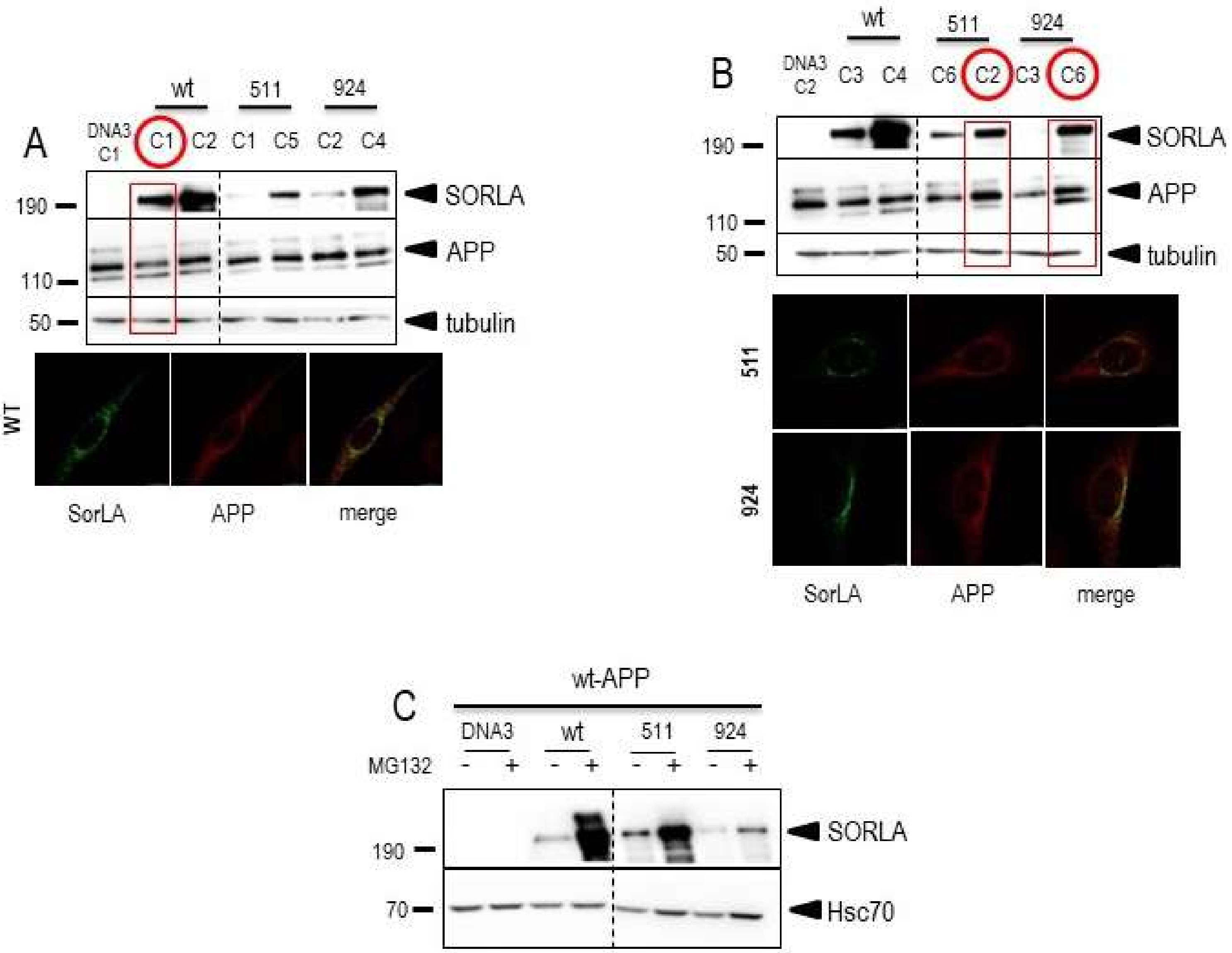

Supplementary figure 1. **Expression and fate of wt-SorLA and its mutants in stably transfected CHO cells**. Red circles indicated the selected stable clones for further analyses

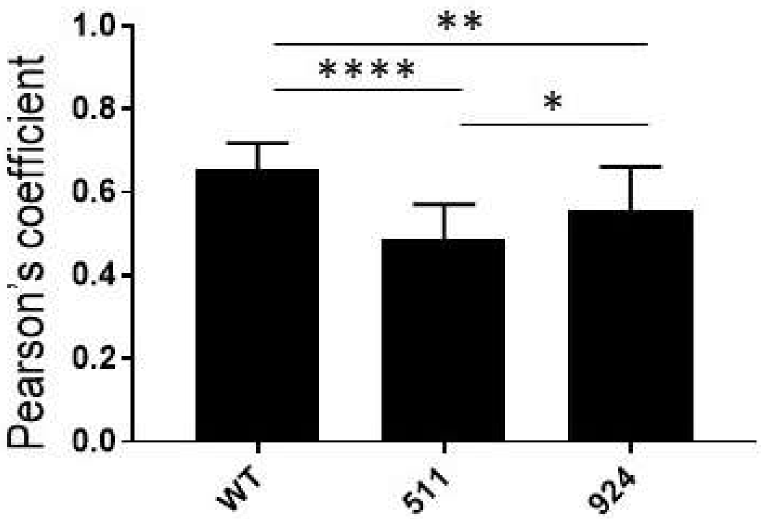

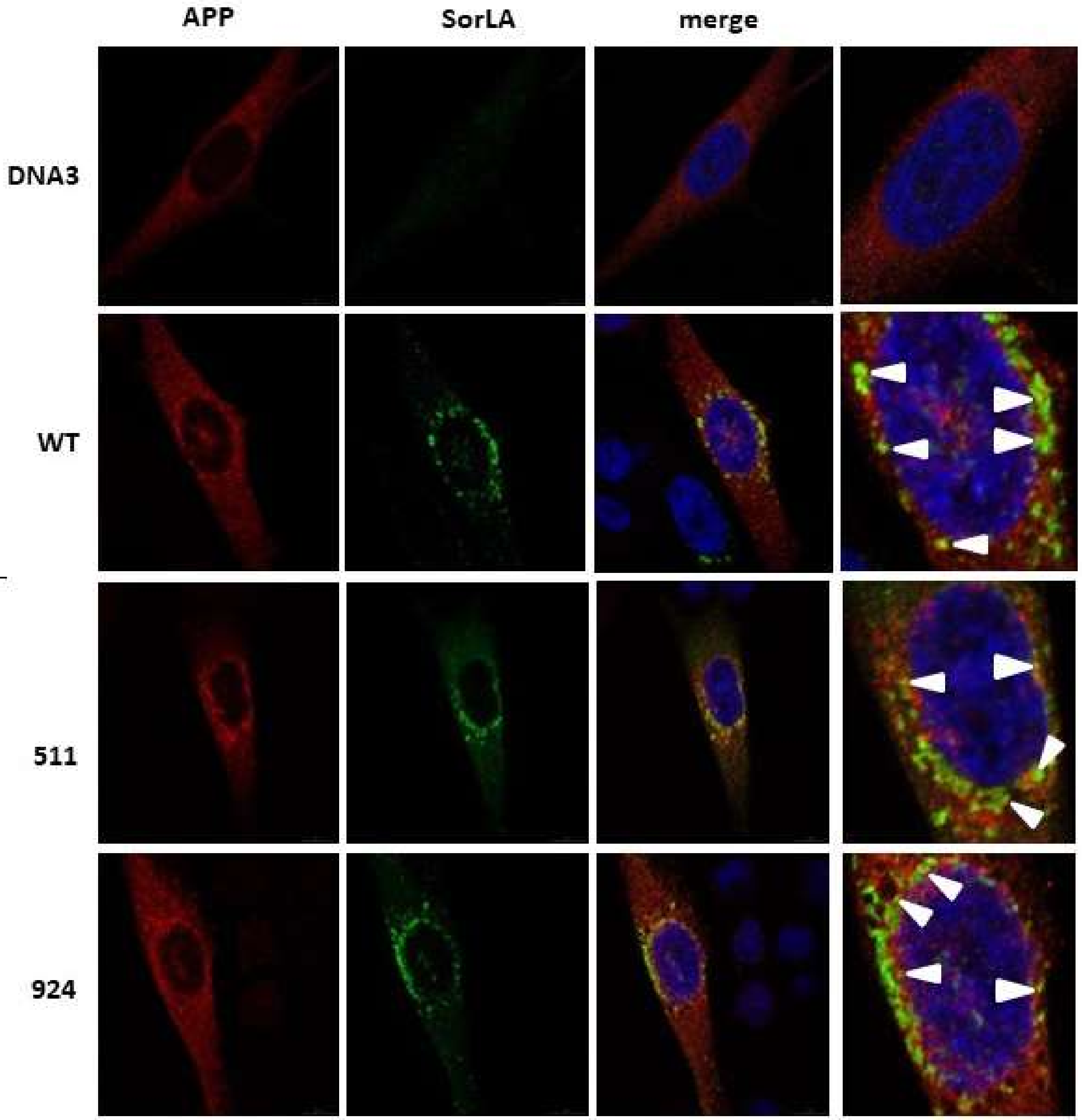

Supplementary figure 2. Immunohistochemical analysis of the co-localization of wt-APP and wt-SorlA of SorLA mutants in CHO stably transfected cells. Red label = APP, green label = SorLA, yellow label = merge, arrows indicate colocalization points

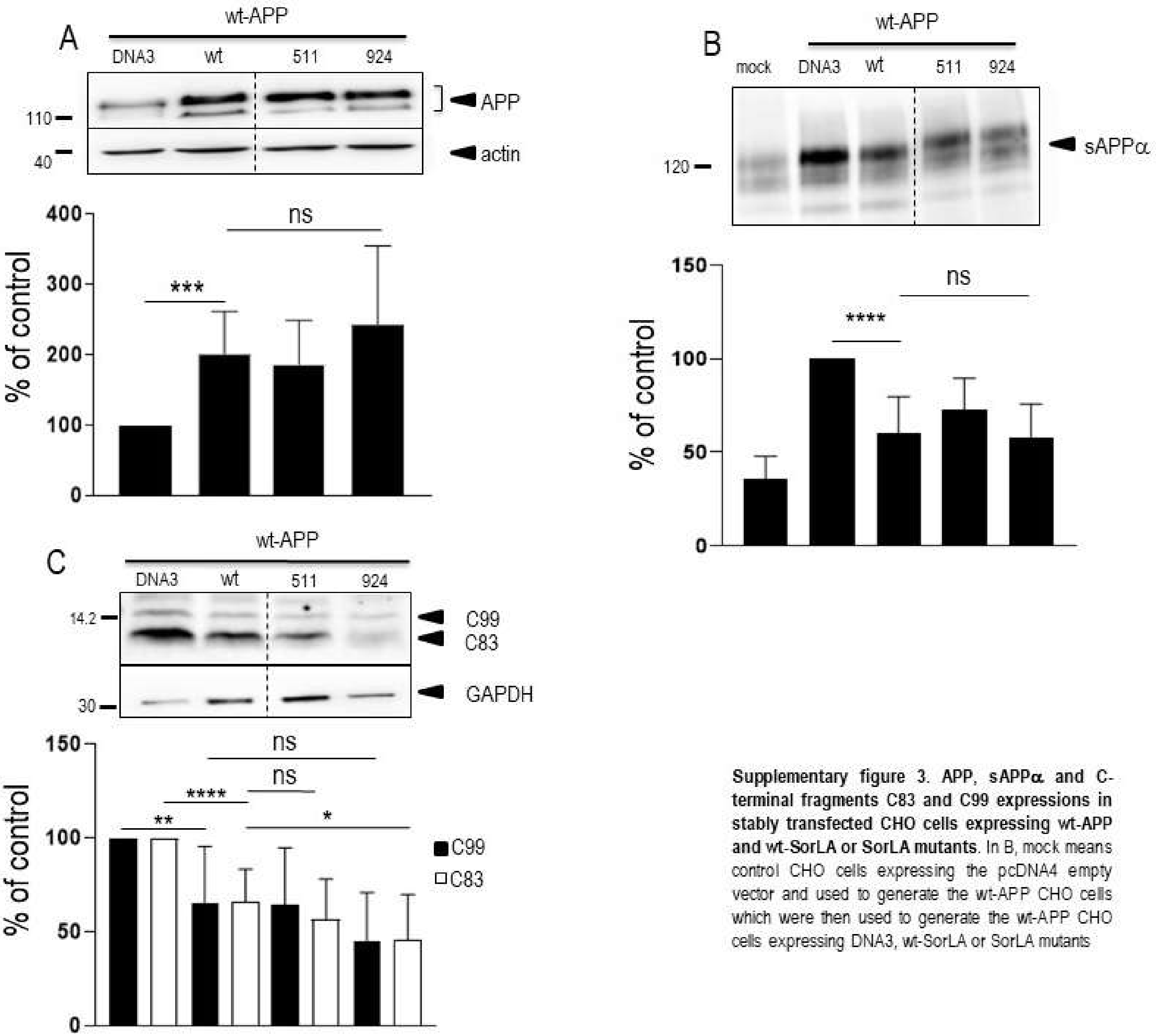

Supplementary figure 3. APP, sAPPα and C-terminal fragments C83 and C99 expressions in stably transfected CHO cells expressing wt-APP and wt-SorLA or SorLA mutants. In B, mock means control CHO cells expressing the pcDNA4 empty vector and used to generate the wt-APP CHO cells which were then used to generate the wt-APP CHO cells expressing DNA3, wt-SorLA or SorLA mutants

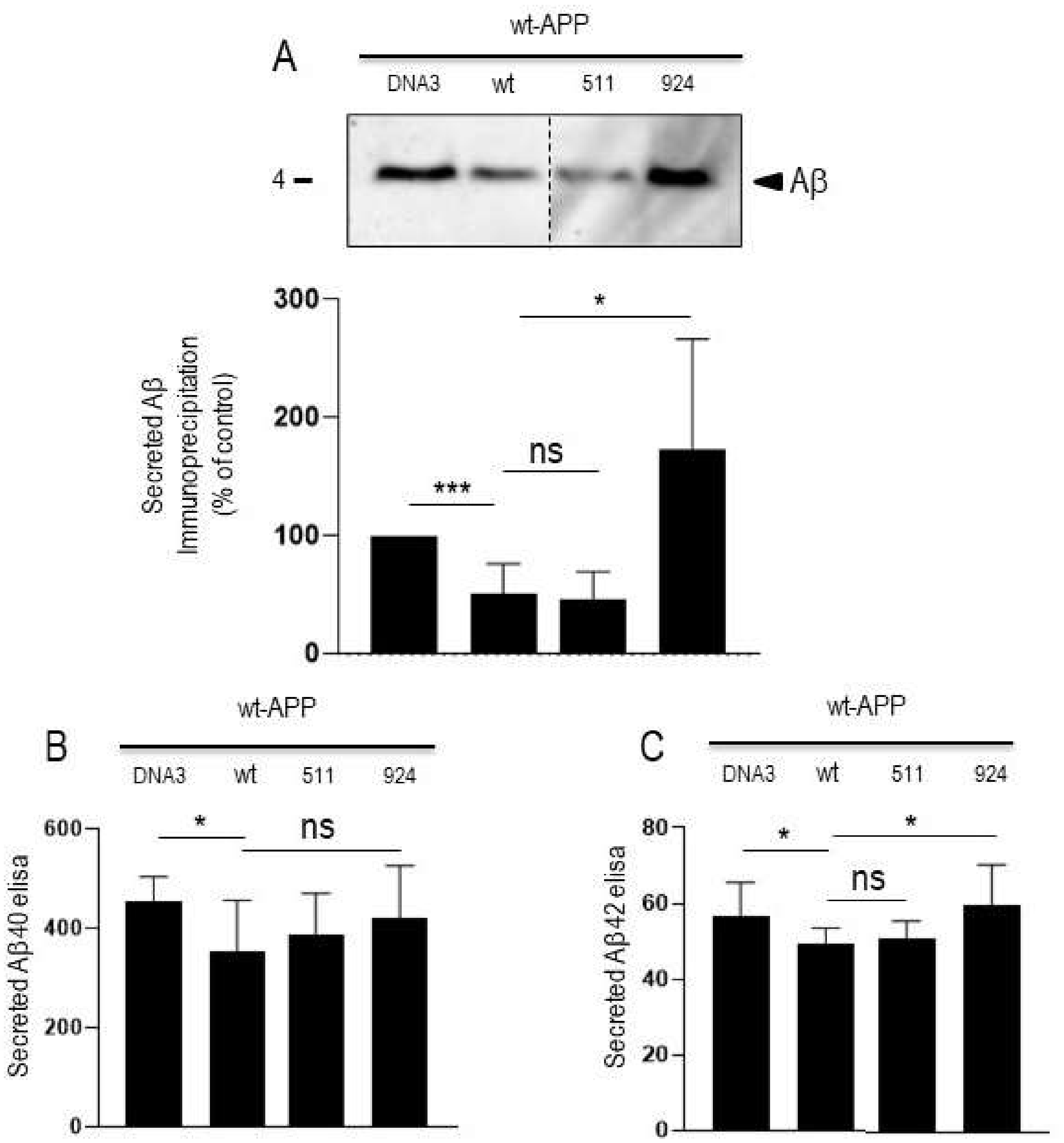

Supplementary figure 4. Secreted Aβ peptides produced by stably transfected CHO cells expressing wt-APP and wt-SorlA or SorLA mutants. In A, total Aβ was analysed by immunoprecipitation. In B and C, cells were analysed for Aβ40 and Aβ42 by ELISA.
