# Supplementary figures and images for: Potentially Pathogenic SORL1 Mutations Observed in Autosomal-Dominant Cases of Alzheimer’s Disease Do Not Modulate APP Physiopathological Processing
Source: Cells. 2023 Dec 8;12(24):2802. doi: 10.3390/cells12242802 (PMC10742224; doi:10.3390/cells12242802)

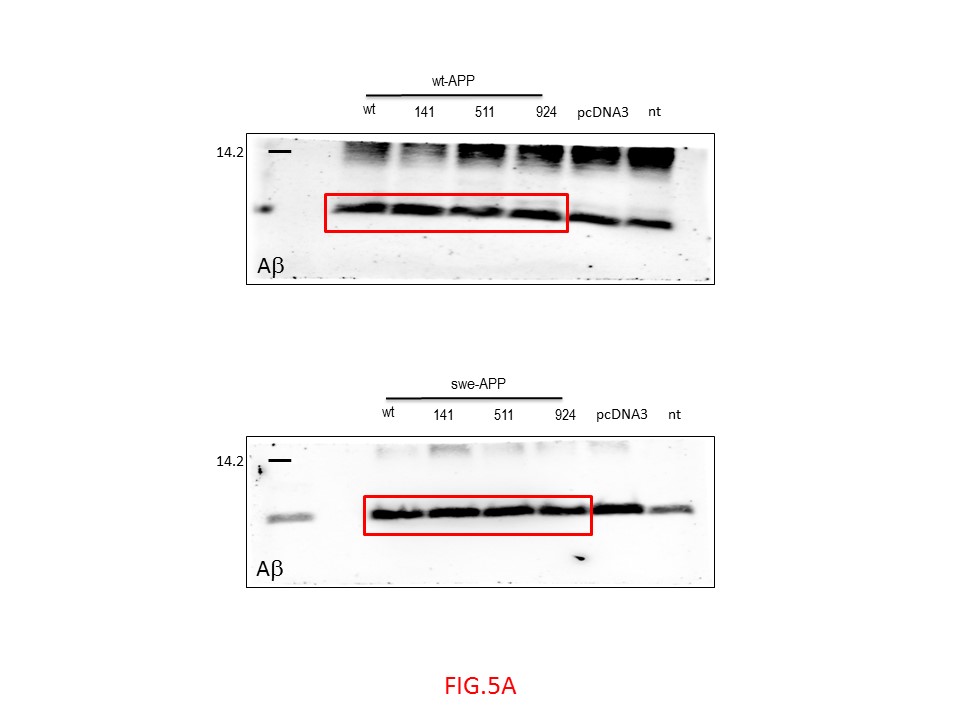

Supplement: Supplementary file 1 [file cells-12-02802-s001.zip › cells-2665629-SM/Full Gel/Figure S10-Full Gel of FIG.5A.JPG]

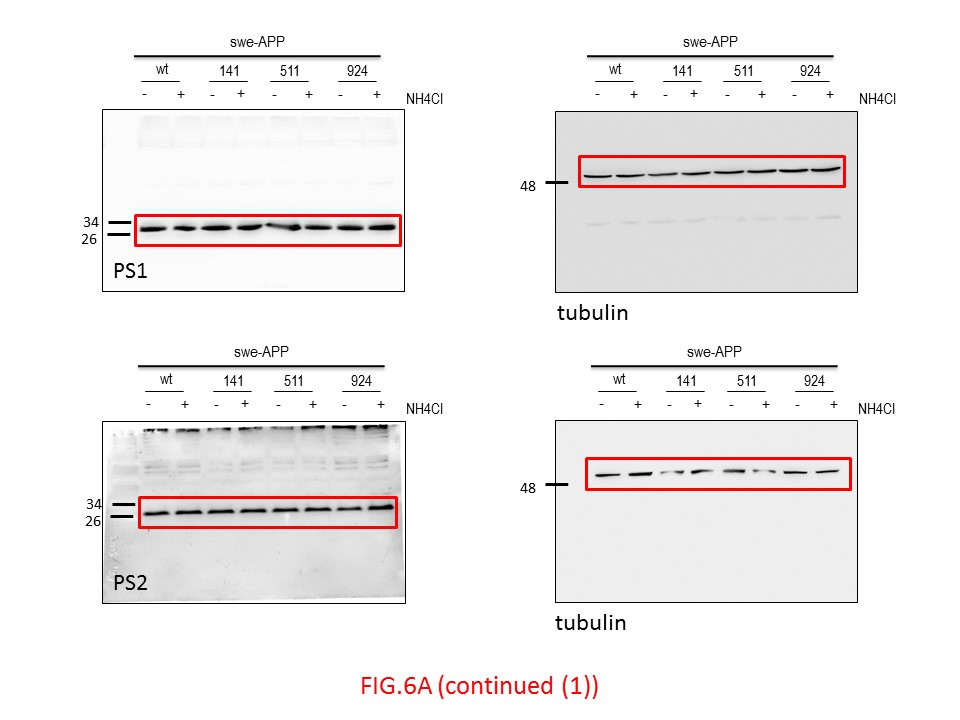

Supplement: Supplementary file 1 [file cells-12-02802-s001.zip › cells-2665629-SM/Full Gel/Figure S11-Full Gel of FIG.6A(1).JPG]

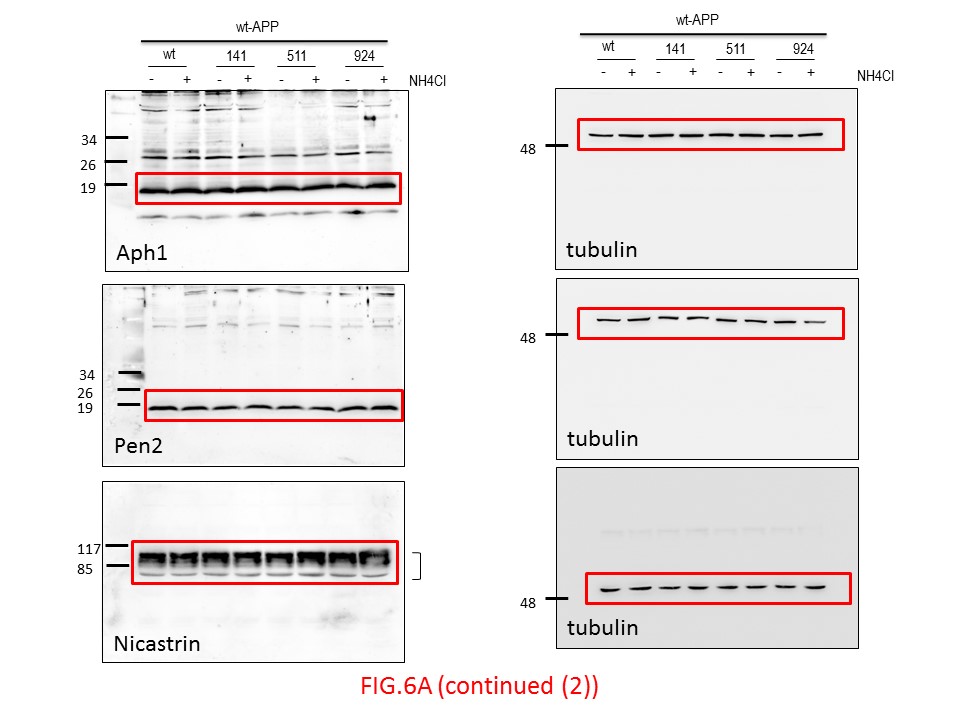

Supplement: Supplementary file 1 [file cells-12-02802-s001.zip › cells-2665629-SM/Full Gel/Figure S12-Full Gel of FIG.6A(2).JPG]

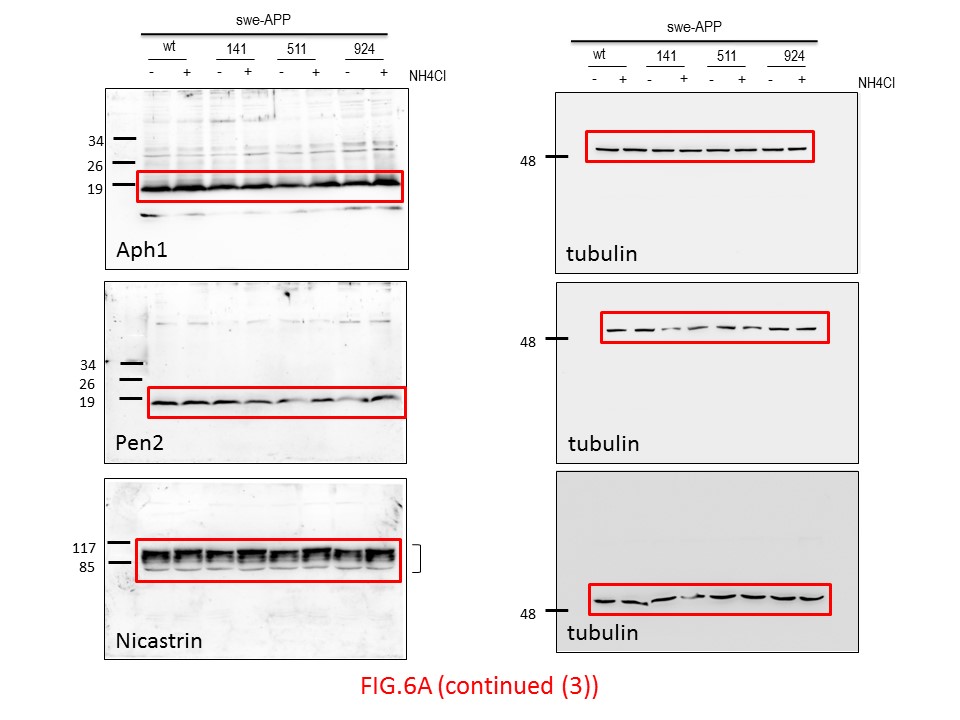

Supplement: Supplementary file 1 [file cells-12-02802-s001.zip › cells-2665629-SM/Full Gel/Figure S13-Full Gel of FIG.6A(3).JPG]

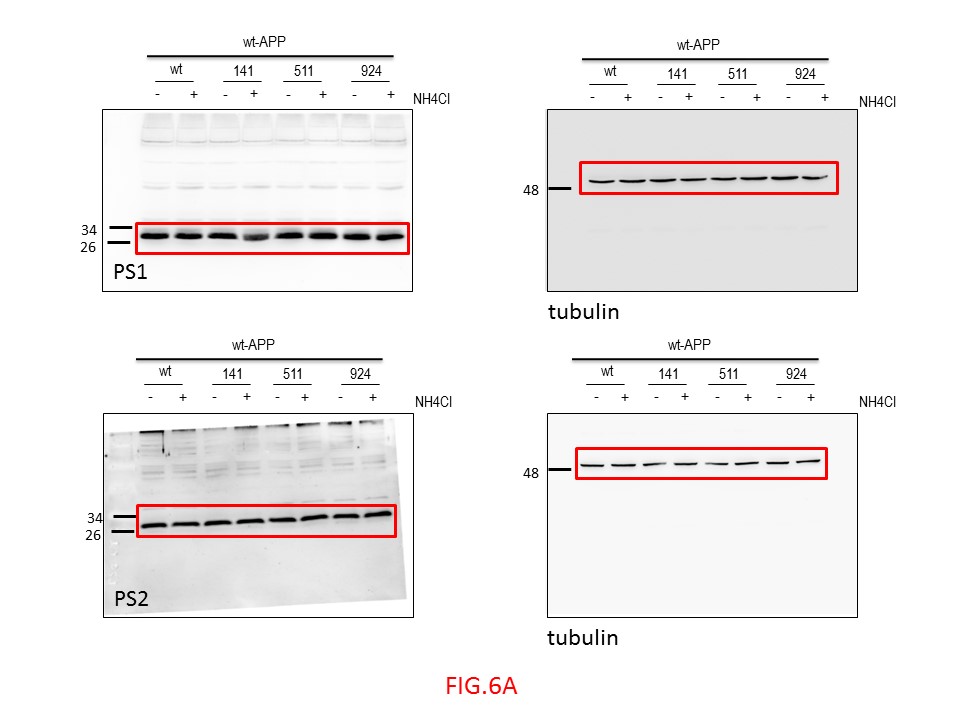

Supplement: Supplementary file 1 [file cells-12-02802-s001.zip › cells-2665629-SM/Full Gel/Figure S14-Full Gel of FIG.6A.JPG]

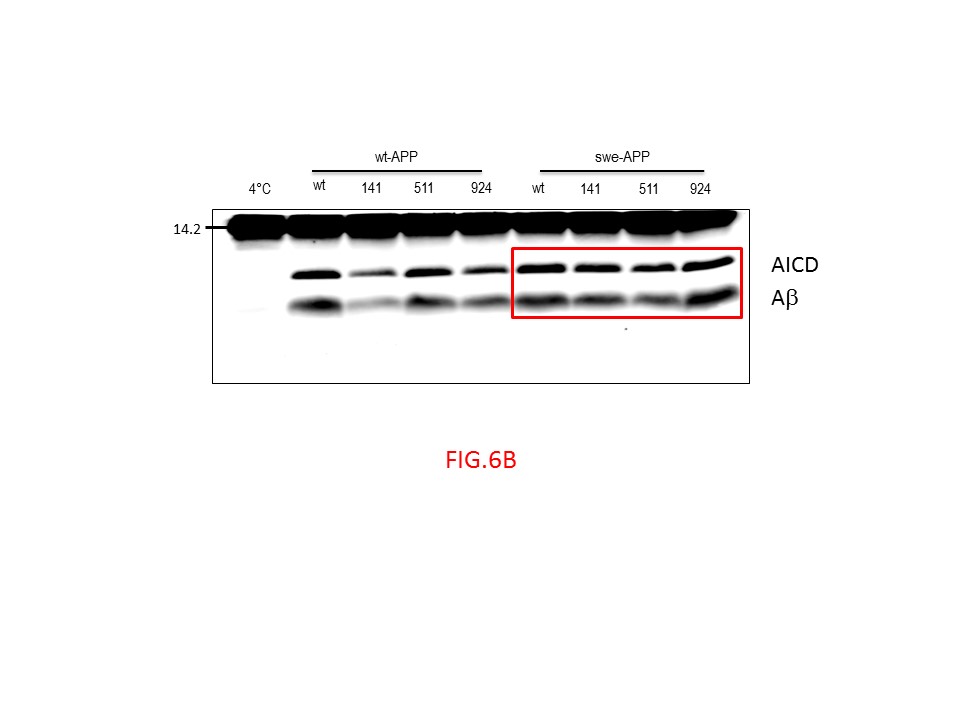

Supplement: Supplementary file 1 [file cells-12-02802-s001.zip › cells-2665629-SM/Full Gel/Figure S15-Full Gel of FIG.6B.JPG]

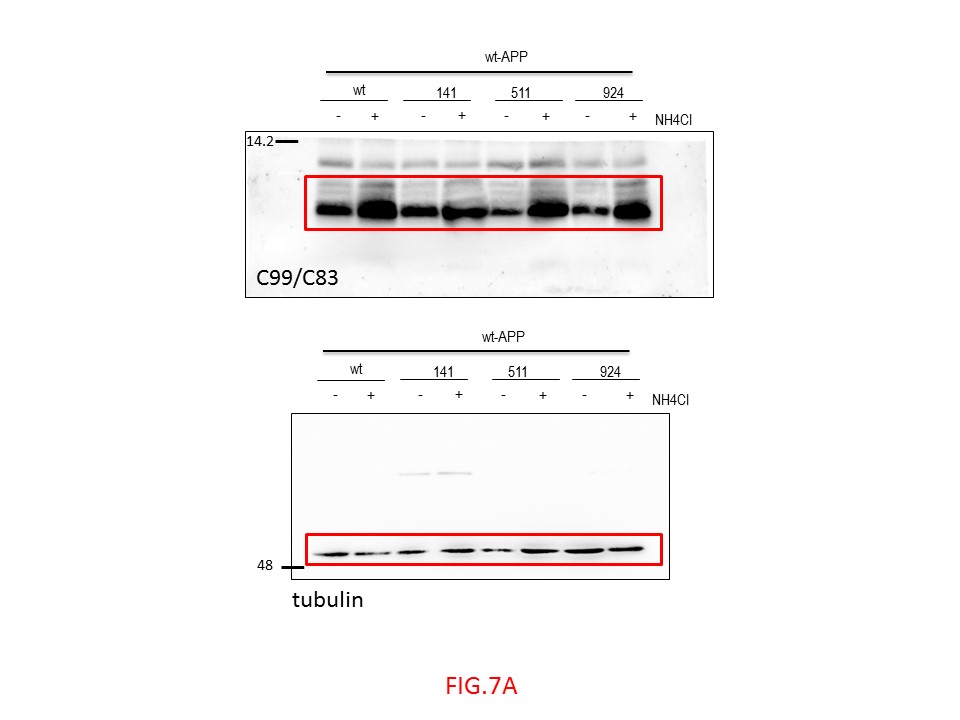

Supplement: Supplementary file 1 [file cells-12-02802-s001.zip › cells-2665629-SM/Full Gel/Figure S16-Full Gel of FIG.7A.JPG]

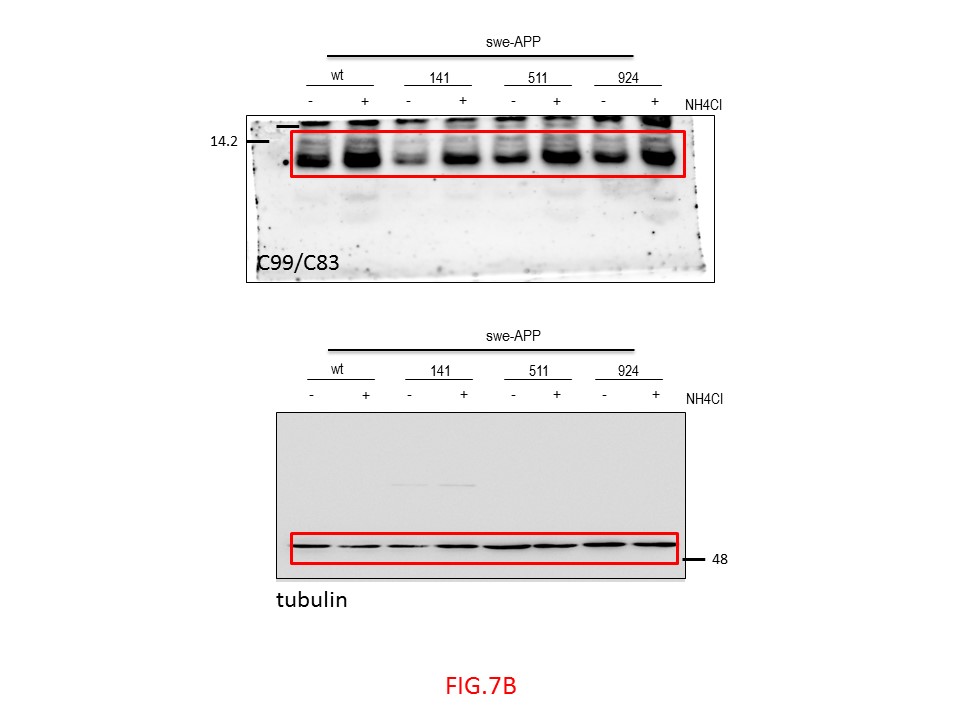

Supplement: Supplementary file 1 [file cells-12-02802-s001.zip › cells-2665629-SM/Full Gel/Figure S17-Full Gel of FIG.7B.JPG]

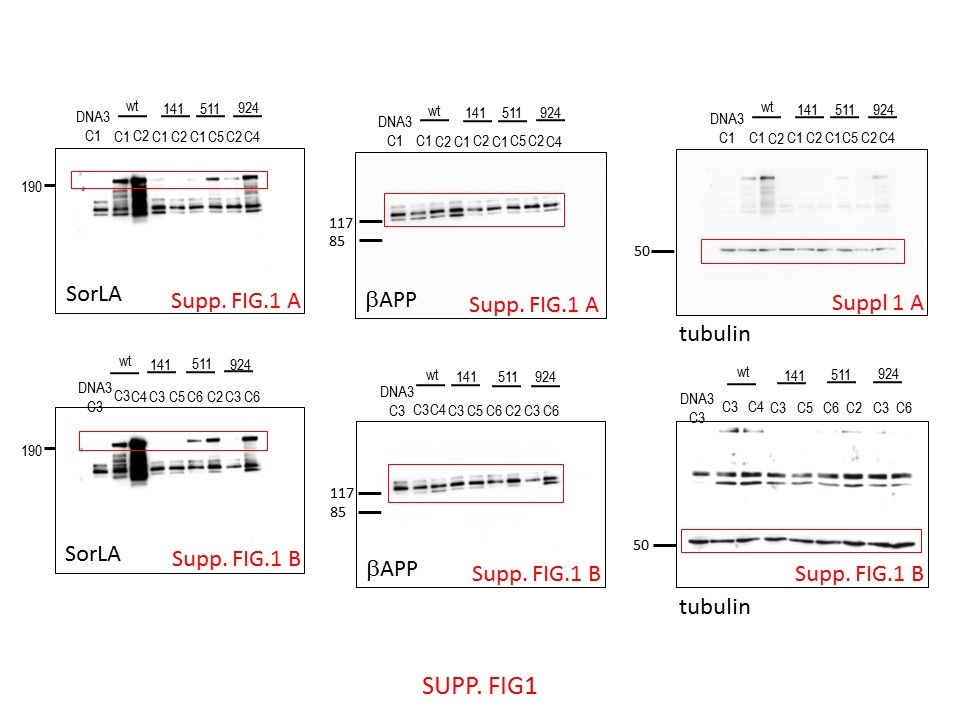

Supplement: Supplementary file 1 [file cells-12-02802-s001.zip › cells-2665629-SM/Full Gel/Figure S18-Full Gel of SUPP. FIG.1.JPG]

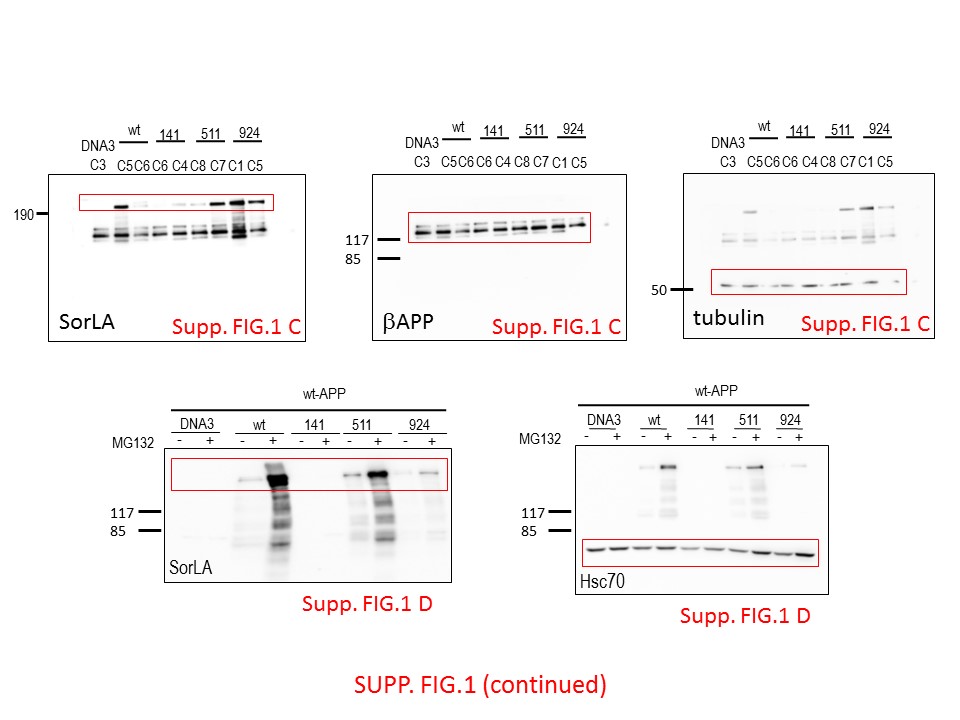

Supplement: Supplementary file 1 [file cells-12-02802-s001.zip › cells-2665629-SM/Full Gel/Figure S19-Full Gel of SUPP. FIG.1 continued.JPG]

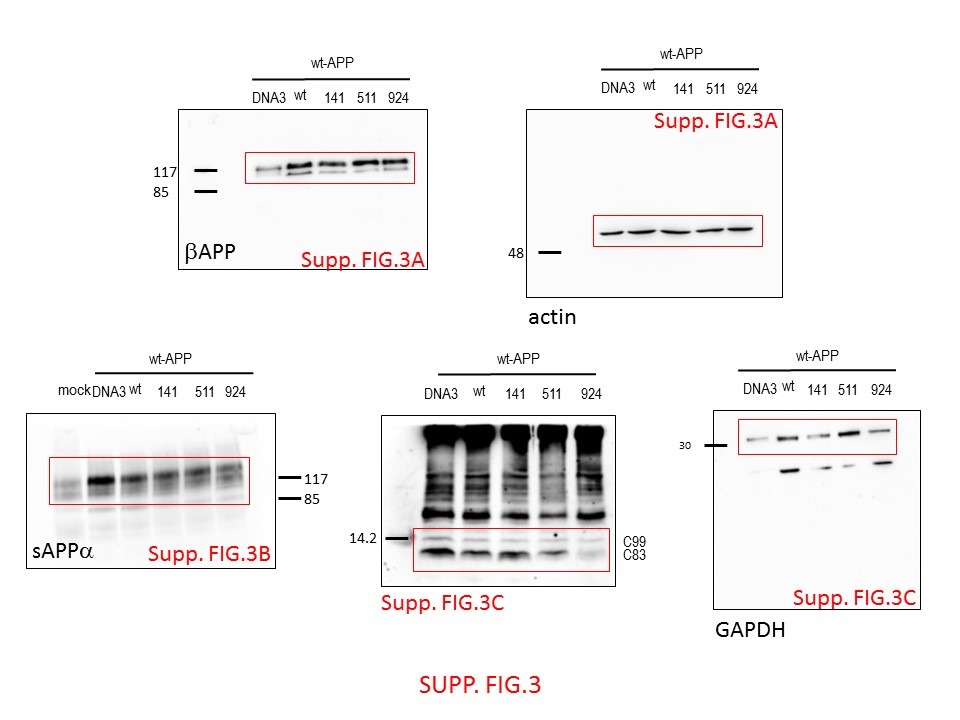

Supplement: Supplementary file 1 [file cells-12-02802-s001.zip › cells-2665629-SM/Full Gel/Figure S20-Full Gel of SUPP. FIG.3.JPG]

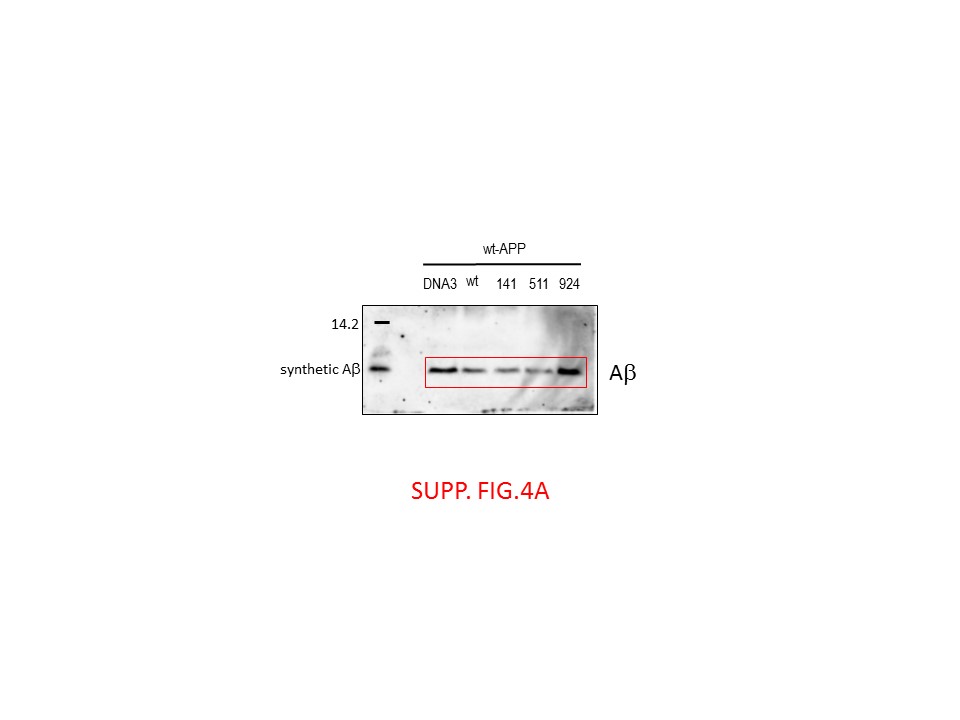

Supplement: Supplementary file 1 [file cells-12-02802-s001.zip › cells-2665629-SM/Full Gel/Figure S21-Full Gel of SUPP. FIG.4A.JPG]

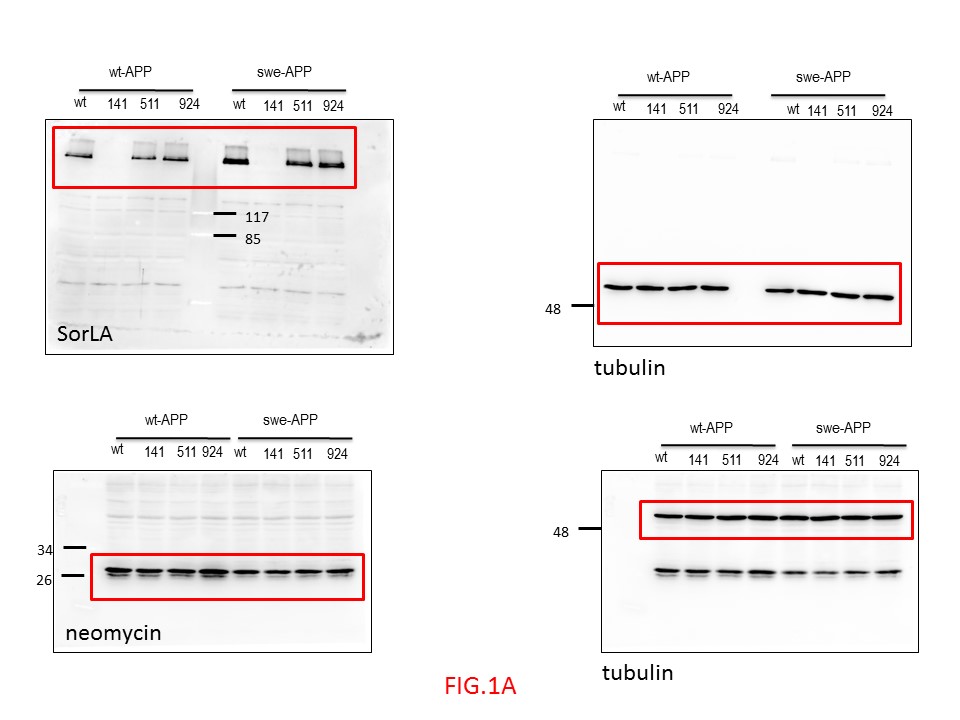

Supplement: Supplementary file 1 [file cells-12-02802-s001.zip › cells-2665629-SM/Full Gel/Figure S5-Full Gel of FIG.1A.JPG]

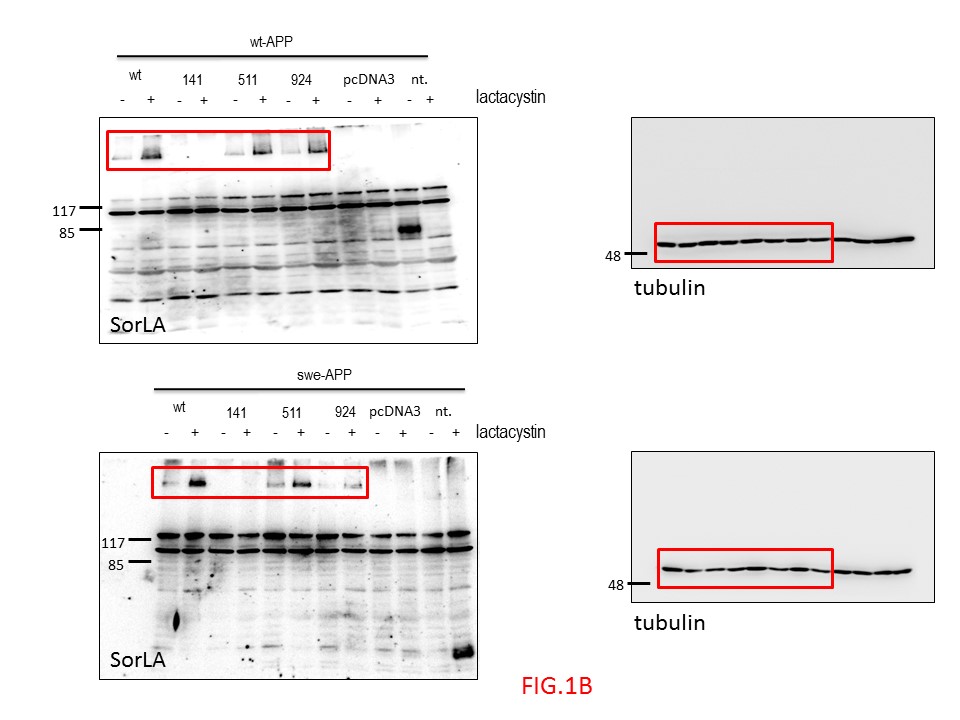

Supplement: Supplementary file 1 [file cells-12-02802-s001.zip › cells-2665629-SM/Full Gel/Figure S6-Full Gel of FIG.1B.JPG]

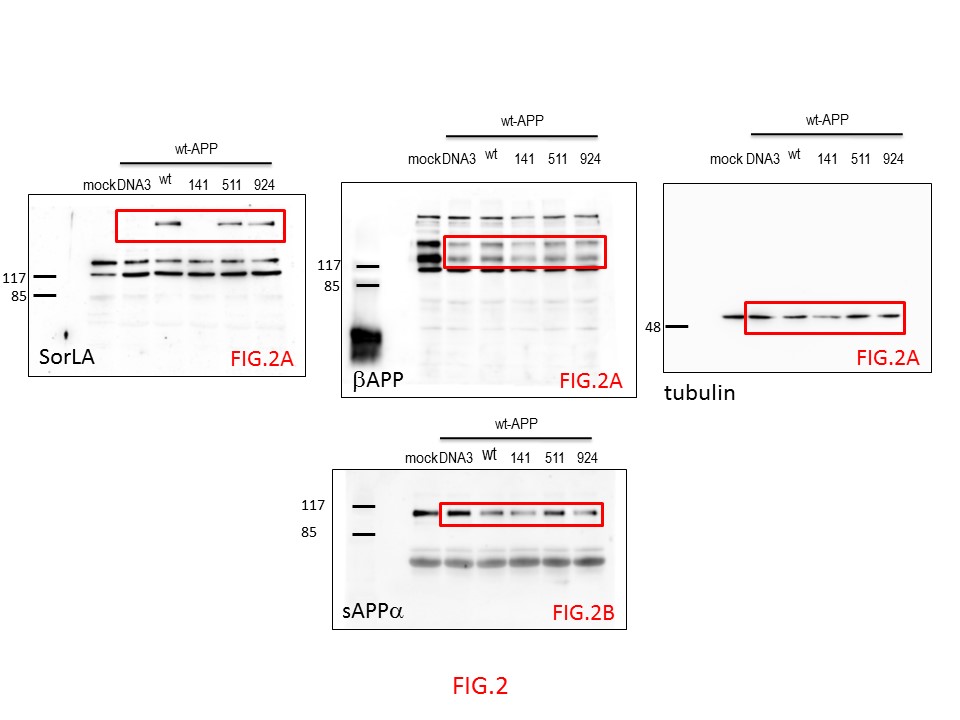

Supplement: Supplementary file 1 [file cells-12-02802-s001.zip › cells-2665629-SM/Full Gel/Figure S7-Full Gel of FIG.2.JPG]

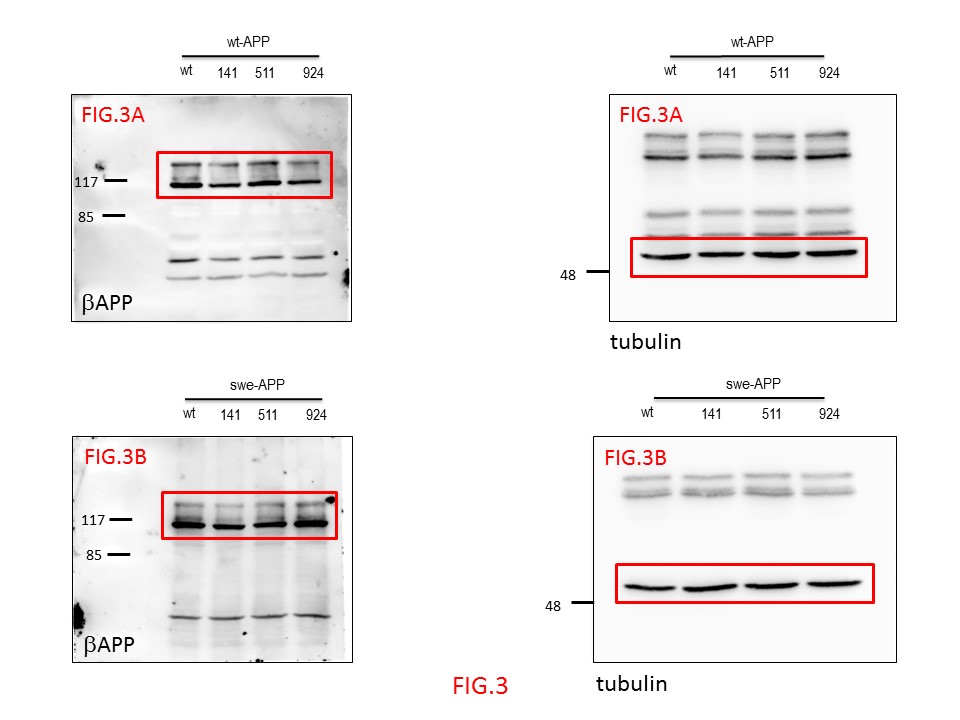

Supplement: Supplementary file 1 [file cells-12-02802-s001.zip › cells-2665629-SM/Full Gel/Figure S8-Full Gel of FIG.3.JPG]

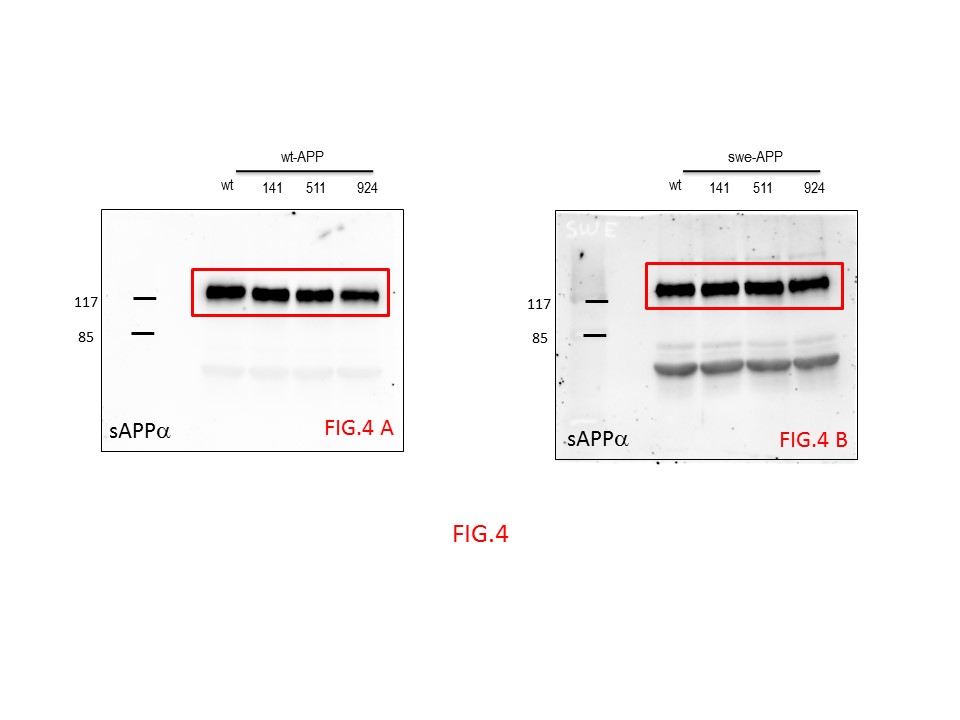

Supplement: Supplementary file 1 [file cells-12-02802-s001.zip › cells-2665629-SM/Full Gel/Figure S9-Full Gel of FIG.4.JPG]
